# Surgical revascularizations for pediatric moyamoya: a systematic review, meta-analysis, and meta-regression analysis

**DOI:** 10.1007/s00381-023-05868-6

**Published:** 2023-02-08

**Authors:** Keng Siang Lee, John J. Y. Zhang, Sanjay Bhate, Vijeya Ganesan, Dominic Thompson, Greg James, Adikarige Haritha Dulanka Silva

**Affiliations:** 1 Department of Neurosurgery, Great Ormond Street Hospital for Children, London, UK; 2 Great Ormond Street Institute of Child Health, University College London, London, UK; 3 Department of Neurosurgery, King’s College Hospital, London, UK; 4 Department of Basic and Clinical Neurosciences, Maurice, Wohl Clinical Neuroscience Institute, Institute of Psychiatry, Psychology and Neuroscience, King’s College London, London, UK; 5 Bristol Medical School, University of Bristol, Bristol, UK; 6 Department of Neurosurgery, National Neuroscience Institute, Singapore, Singapore; 7 Department of Paediatric Neurology, Great Ormond Street Hospital for Children, London, UK

**Keywords:** Neurosurgery, Pediatric, Moyamoya disease, Moyamoya syndrome, Bypass, Revascularization

## Abstract

**Introduction:**

There is no clear consensus regarding the technique of surgical revascularization for moyamoya disease and syndrome (MMD/MMS) in the pediatric population. Previous meta-analyses have attempted to address this gap in literature but with methodological limitations that affect the reliability of their pooled estimates. This meta-analysis aimed to report an accurate and transparent comparison between studies of indirect (IB), direct (DB), and combined bypasses (CB) in pediatric patients with MMD/MMS.

**Methods:**

In accordance with PRISMA guidelines, systematic searches of Medline, Embase, and Cochrane Central were undertaken from database inception to 7 October 2022. Perioperative adverse events were the primary outcome measure. Secondary outcomes were rates of long-term revascularization, stroke recurrence, morbidity, and mortality.

**Results:**

Thirty-seven studies reporting 2460 patients and 4432 hemispheres were included in the meta-analysis. The overall pooled mean age was 8.6 years (95% CI: 7.7; 9.5), and 45.0% were male. Pooled proportions of perioperative adverse events were similar between the DB/CB and IB groups except for wound complication which was higher in the former group (RR = 2.54 (95% CI: 1.82; 3.55)). Proportions of post-surgical Matsushima Grade A/B revascularization favored DB/CB over IB (RR = 1.12 (95% CI 1.02; 1.24)). There was no significant difference in stroke recurrence, morbidity, and mortality. After meta-regression analysis, year of publication and age were significant predictors of outcomes.

**Conclusions:**

IB, DB/CB are relatively effective and safe revascularization options for pediatric MMD/MMS. Low-quality GRADE evidence suggests that DB/CB was associated with better long-term angiographic revascularization outcomes when compared with IB, although this did not translate to long-term stroke and mortality benefits.

**Supplementary Information:**

The online version contains supplementary material available at 10.1007/s00381-023-05868-6.

## Introduction

Moyamoya disease (MMD) or syndrome (MMS) refers to an abnormal progressive steno-occlusive disorder at the distal internal carotid artery (ICA) [1]. Patients are at high risk for transient ischemic stroke (TIA) or stroke. It is asserted that surgical revascularization provides better outcomes for these patients than medical treatment alone. In pediatric patients, the goal of surgery is to augment cerebral blood flow and reduce the risk of ischemic events [2].

Revascularization techniques can be direct, indirect, or combined. Direct bypass (DB) is accomplished by anastomosing extracranial vessels to intracranial vessels (EC-IC bypass), most often the superficial temporal artery (STA) to the middle cerebral artery (MCA) (STA-MCA bypass) [3, 4]. Indirect bypass (IB) has many variations but is generally accomplished by incorporating well-vascularized tissue usually from external carotid artery sources onto the surface of the brain to promote angiogenesis and neovascularization, rather than by direct anastomosis [5–9]. Unlike DB, IB begins to alter the cerebral blood flow only after angiogenesis has taken place, the timescale for which is unpredictable [3]. A combined bypass (CB) utilizes both techniques simultaneously to maximize the effect of short-term and long-term revascularization [3].

There is currently no definite consensus regarding the technique of surgical revascularization in pediatric MMD/MMS [1, 2]. Existing meta-analyses have elegantly attempted to address this controversy in pediatric patients [10, 11]. However, in these studies, repeated patient populations from the same institutions within overlapping time intervals were included [10, 11]. This methodological flaw overstates sample size and number of events, leading to an artificially exaggerated precision in their pooled estimate [12]. In addition, several included primary studies in these meta-analyses had not distinguished outcomes based on the type of bypass nor specifically for children, and hence it was unclear how these meta-analyses were able to distinguish between the techniques or population. This meta-analysis aimed to mitigate against previous methodological limitations and report an accurate and transparent comparison between studies of IB, DB, and CB in pediatric patients with MMD/MMS.

## Methods

This review was conducted according to the Preferred Reporting Items for Systematic Reviews and Meta-Analyses (PRISMA) guidelines [13]. The protocol was registered on the PROSPERO international prospective register (CRD42022365524).

### Outcomes

The primary outcome included any reported perioperative adverse events within 30 days after bypass surgery. This included wound complications, seizures, cerebrospinal fluid (CSF) leak extra-axial hemorrhage, TIA, stroke, and death.

Secondary outcomes included modified Rankin score (mRS), long-term stroke, mortality risk, and degree of angiographic revascularization at last follow-up. The degree of revascularization was graded according to Matsushima’s classification of the proportion of arterial territory (Grade A > 2/3, Grade B = 1/3 to 2/3, Grade C < 1/3) [3].

When angiographic assessment in the primary studies was graded according to a 3-grade classification (poor, moderate, or good), or 4-grade classification (none, poor, medium, or extensive) [14], we classified “good” and “extensive” as Grade A, “moderate” and “medium” as Grade B, and “poor” or “none” as Grades C.

### Search strategy

Searches of three electronic databases were undertaken: Ovid Medline, Ovid Embase, and Cochrane Central Register of Controlled Trials (CENTRAL). Searches were performed in each database from its inception until 7 October 2022. The full search strategy is presented in Supplementary Table 1.

### Eligibility criteria

Articles were selected for inclusion if they were a primary interventional or observational study evaluating the effectiveness and safety of revascularization surgeries in pediatric MMD/MMS. The review included studies on exclusively pediatric patients (< 18 years). Studies that had evaluated both pediatric and adult MMD/MMS but reported outcomes specific to the pediatric population were included. Studies that had evaluated various revascularization techniques but reported outcomes specific for the particular technique were included.

The review excluded narrative and systematic reviews, editorials, commentaries, opinion papers, letters, education papers, conference abstracts, protocols, reports, theses, or book chapters as they were unlikely to contain sufficient detail about the effectiveness and safety of both treatments.

### Study selection

All titles and abstracts were screened against the pre-defined eligibility criteria developed independently by two reviewers (KSL and JJYZ). A full list of inclusion and exclusion criteria of studies is stated in Supplementary Table 2. Disagreements were resolved by discussion, and where agreement could not be reached, the senior reviewer assisted with decision-making (AHDS). Agreement among the reviewers was evaluated using Cohen’s kappa [15].

The institutions and data collection period were scrutinized to avoid multiple counting. In the event of multiple publications analyzing the same cohort of patients/hemispheres, the publication that reported the largest patient data with the most relevant outcomes was used for evaluation.

### Data extraction

A pro forma was developed and piloted to extract data on the following variables to ensure standardization and consistency in this process: (1) study details; (2) study design; (3) participant demographics; (4) country, institution, and data collection period; (5) selection criteria; (6) treatment and control; (7) indication for treatment; and (8) results.

### Risk of bias assessment

The quality of included studies was assessed using the Joanna Briggs Institute (JBI) checklist for cohort studies and case series [16]. KSL and JJYZ assessed the quality of all included studies and discussed discrepancies until consensus was reached.

### Statistical analysis

Meta-analyses of primary end points were performed assuming the random effects model to account for heterogeneity within and between individual studies [16].

We analyzed both DB and CB as a single cohort compared with IB. The rationale is that in CB, patients undergo a direct and an indirect component of the revascularization in the same setting. The direct component would afford an immediate increase in cerebral perfusion, while the indirect collateralization would take months to a year to form [3]. As reported denominators were heterogenous, analyses by both patients and hemispheres were performed whenever possible. To obtain risk ratios (RRs) from reported binary outcomes between DB/CB and IB, a pairwise meta-analysis was conducted using the Mantel–Haenszel method, using the Paule-Mandel estimator. Overall pooled proportions were computed using the generalized linear mix model (GLMM) [16]. Knapp-Hartung adjustments were used to calculate the 95% confidence intervals (CIs) around the pooled effect to reduce the risk of a Type 1 error.

For the pooling of means of numerical variables, we computed missing means and standard deviations (SDs) from medians, ranges, and interquartile ranges (IQRs) using the methods proposed by Hozo et al. and Wan et al. [17, 18].

The *I*^2^ statistic was used to present inter-study heterogeneity, where *I*^2^ ≤ 30%, between 30 and 50%, between 50 and 75%, and ≥ 75% were considered to indicate low, moderate, substantial, and considerable heterogeneity, respectively [16]. *P* values for the *I*^2^ statistic were derived from the chi-squared distribution of Cochran’s *Q* test.

Summary-level meta-regression was performed using the mixed-effects model after computation of the SD of Freeman-Tukey double arcsine transformed proportions, to identify predictors of perioperative TIA, stroke, long-term revascularization, stroke, and mortality. Predictors were year of publication, age, presence of MMS, presence of sickle cell disease (SCD), neurofibromatosis (NF1), and Down syndrome, in accordance with the literature [5, 7, 19, 20]. Summary-level meta-regression was additionally performed using a mixed-effect meta-analysis model by the GLMM method, as a sensitivity analysis to examine the robustness of the former approach.

The publication bias of studies was assessed visually using funnel plots and quantitatively using Egger’s regression test [16]. The GRADE approach was used to evaluate the quality of evidence for each outcome.

All statistical analyses were performed using R software version 4.2.1 (R Foundation for Statistical Computing, 2022), with the package *meta. P* values less than 0.05 were considered statistically significant.

## Results

### Study selection and characteristics

As expected, a substantial number of studies were excluded because they had reported data from the same cohort of patients/hemispheres across overlapping time periods. These were commonly from large high-volume institutions such as Beijing Tiantan Hospital [21–25], Boston Children’s Hospital [5–9, 20, 26–30], and Seoul National University Children’s Hospital [31–34]. Consequently, only one publication that reported the largest patient data with the most relevant outcomes was included in our analysis.

**Fig. 1:**
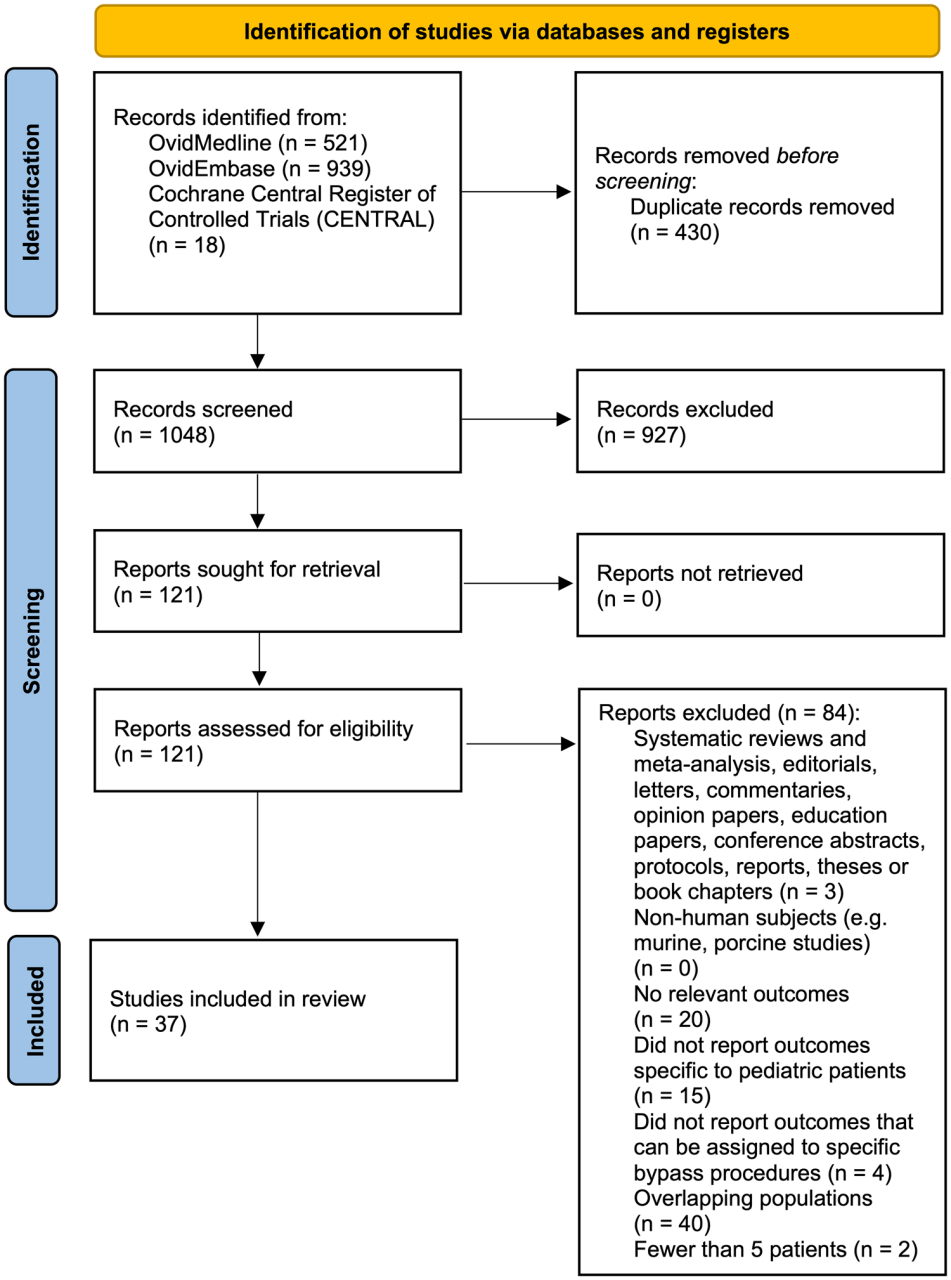
PRISMA flow diagram for studies included and excluded

Thirty-seven studies met the eligibility criteria for inclusion in our systematic review and meta-analysis (Fig. 1) [2–4, 19, 35–67]. The reliability of the study selection was substantial at both the title and abstract (Cohen’s *κ* = 0.86) and the full-text review stages (Cohen’s *κ* = 1.00) [15].

All included studies were retrospective observational studies—eight cohort studies and 29 case series. A total of 37 studies reporting 2460 pediatric patients were included. Only 36 studies had reported the number of hemispheres, and the total hemisphere count was 4432. Thirty-two studies reported outcomes of IB. Of these, 31 studies reported the number of hemispheres in the IB group (3524) and all 34 reported the number of patients (2227). Seventeen studies reported outcomes of DB/CB. All 17 studies reported the number of hemispheres in the DB/CB group (905) whilst only 10 reported the number of patients (358). Eleven studies compared outcomes between the IB and DB/CB groups (Table 1).

**Table 1:** Summary of included studies

First author and year	Country	Study design	Institution	Study period	Total hemispheres/surgeries, *n*	Total patients, *n*	Type of revascularization	Etiology, *n*	Imputed mean age at surgery (SD), year*	Male, *n* (%)
Alamri et al. (2019) [35]	UK	Case series	King’s College Hospital	2007 to 2015	9	8	IB–EDAS	8 MMMS, 8 SCD	12.7 (2.6)	4 (50.0)
Araki et al. (2022) [36]	Japan	Cohort study	Nagoya University Graduate School of Medicine	2005 to 2021	39	21	IB–EDMS, DB–STAMCA	21 MMD	3.6 (1.1)	NR
Bao et al. (2015) [37]	China	Case series	People's Liberation Army (PLA) Hospital	2002 to 2010	512	288	IB–EDAS	288 MMD	9.9 (4.9)	146 (50.7)
Blauwblomme et al. (2017) [38]	France	Case series	Necker Hospital	1999 to 2015	108	64	IB–MBH	32 MMMS, 7 SCD, 7 DS, 8 NF1	9.1 (4.1)	33 (51.6)
Chen et al. (2018) [39]	China	Case series	The Third Affiliated Hospital	2002 to 2015	18	10	IB–Pial synangiosis	MMD	8.5(1.9)	2 (20.0)
Czabanka et al. (2009) [40]	Germany	Case series	Charité-Universitätsmedizin Berlin	NR	20	10	IB–EMS, DB–STAMCA	MMD	8.4 (6.4)	4 (40.0)
Darwish et al. (2005) [41]	Australia	Case series	Royal Alexandra Hospital for Children	1982 to 2004	21	13	IB–EDAS, EMS, DB–STAMCA	4 MMMS, 3 NF1, 1 cranial radiation	6.1 (3.9)	5 (38.5)
De Oliveira et al. (2009) [42]	Mexico	Case series	Ribeirao Preto Medical School	2003 to 2008	14	7	IB–MBH	3 MMS, 3DS	8.4 (3.7)	2 (28.6)
Deng et al. (2021) [43]	China	Cohort study	Beijing Tiantan Hospital	2010 to 2019	533	336	IB–EDAS, MBH, DB–STAMCA	MMD	9.6 (3.7)	NR
Funaki et al. (2014) [44]	Japan	Case series	Kyoto University Graduate School of Medicine	1978 to 2003	114	58	DB–STAMCA	MMD	6.4 (4.3)	24 (41.4)
Furtado et al. (2021) [45]	India	Case series	MS Ramaiah Medical College and Hospital	2006 to 2019	50	28	IB–EDAS	MMD	NR	NR
Gadgil et al. (2018) [46]	USA	Case series	Texas Children’s Hospital	1997 to 2016	169	102	IB–EDAS, dural inversion	60 MMS, 43 SCD, 5 DS, 1 NF1, 8 cranial radiation, 1 ACTA2	10.9 (6.8)	46 (45.1)
Goren et al. (2021) [47]	Israel	Case series	Sheba Medical Center Hospital	2000 to 2019	49	27	IB–EDAS, dural inversion	5 MMS, 3 DS, 1 NF1, 1 AS	7.0 (4.7)	16 (59.3)
Griessenauer et al. (2015) [48]	USA	Case series	University of Alabama at Birmingham	2007 to 2014	21	14	IB–EDAS, EMAS	14 MMMS, 14 SCD	15.3 (5.3)	5 (35.7)
Guzman et al. (2009) [49]	USA	Cohort study	Stanford University Medical Center	1991 to 2008	168	96	IB–EDAS, EDMS, DB–STAMCA	16 MMS, 3 DS, 5 NF1, 1 AS	9.8 (4.9)	NR
Ha et al. (2019) [50]	Korea	Case series	Seoul National University Children’s Hospital	1988 to 2012	1283	629	IB–EDAS, MBH	MMMD	7.7 (4.7)	303 (48.2)
Hall et al. (2016) [51]	USA	Case series	Multi-center – Riley Hospital for Children at Indiana University Health and St. Louis Children’s Hospital/Washington University School of Medicine	2000 to 2014	20	12	IB–EDAS, MBH	12 MMS, 12 SCD	NR	8 (66.7)
Ishikawa et al. (1997) [52]	Japan	Cohort study	Hokkaido University School of Medicine, Sapporo, Japan	1988 to 1995	64	34	IB–EDAMS, EDAS, DB–STAMCA	MMD	7.6 (3.6)	15 (44.1)
Isono et al. (2002) [53]	Japan	Case series	Oita Medical University	1983	22	11	IB–EDAMS, EDAS	MMD	6.3 (2.9)	NR
Karasawa et al. (1992) [54]	Japan	Case series	Osaka Neurological Institute	1974 to 1991	196	104	IB–EMS	MMD	NR	NR
Kennedy et al. (2014) [19]	USA	Case series	Columbia University Medical Center/Morgan Stanley Children’s Hospital of New York	1996 to 2012	27	17	IB–pial synangiosis	17 MMS, 17 SCD	NR	8 (47.1)
Kim et al. (2007) [55]	Korea	Cohort study	The Catholic University of Korea College of Medicine, Uijeongbu St. Mary’s Hospital	NR	36	24	IB–EDAMS, EDAS, STAMCA	MMD	NR	12 (50.0)
King et al. (2010) [56]	Canada	Case series	Hospital for Sick Children	1996 to 2008	18	12	IB–pial synangiosis, MBH	12	NR	13
Kuroda et al. (2010) [57]	Japan	Case series	Hokkaido University Graduate School of Medicine	1998 to 2009	47	28	IB–EDAMS, DB–STAMCA	MMD	9.8 (3.8)	7 (25.0))
Matsushima et al. (1998) [3]	Japan	Case series	Kyushu University Graduate School of Medical Sciences	1983 to 1986	72	50	IB–EDAS, EMAS, EMS, DB–STAMCA	MMD	NR	NR
Mirone et al. (2019) [58]	Italy	Case series	Santobono- Pausilipon Children’s Hospital	2007 to 2016	14	10	IB–MBH	6 MMS, 3 NF1, 2 cranial radiation	NR	NR
Morshed et al. (2020) [4]	USA	Cohort study	University of California San Francisco	2006 to 2018	49	26	IB–EDAMS, EDAS, EMS, DB–STAMCA	11 MMD, 1 DS, 4 NF1, 1 cranial radiation, 2 ACTA2	8.5 (4.8)	NR
Ng et al. (2012) [59]	UK	Case series	Great Ormond Street Hospital	1996 to 2010	134	73	IB–EDMS, EDAS, pial synangiosis, DB–STAMCA	27 MMS, 13 SCD, 7 DS, 4 NF1, 10 congenital cardiac abnorality,, 2 renal artery stenosis	8.4 (4.9)	31 (42.5)
Ogiwara and Morota (2012) [60]	Japan	Case series	National Center for Child Health and Development, Tokyo	2003 to 2010	22	12	IB–EDAS, EGS	MMD	6.4 (2.2)	7 (58.3)
Ong et al. (2020) [61]	Singapore	Case series	Multi-center – KK Women’s and Children’s Hospital and the National University Hospital of Singapore	2002 to 2019	23	15	IB–EDAS, EMAS, pial synangiosis, DB–STAMCA	MMD	9.4 (4.7)	5 (33.3)
Rashad et al. (2016) [62]	Japan	Case series	Tohoku University Graduate School of Medicine	2004 to 2015	39	23	IB–EDMS, DB–STAMCA	1 MMMS, 1 NF1	9.4 (4.0)	NR
Sadashiva et al. (2016) [63]	India	Cohort study	National Institute of Mental Health and Neurosciences	2006 to 2014	85	54	IB–E DAMS, DB–STAMCA	MMMD	9.0 (4.7)	25 (46.3)
Sakamoto et al. (1997) [64]	Japan	Case series	Oaska City General Hospital		19	10	IB–EMS, DB–STAMCA	MMD	NR	0 (0.0)
Scott et al. (2004) [2]	USA	Case series	Boston Children’s Hospital	1985 to 2001	271	143	IB–Pial synangiosis	77 MMS, 3 SCD, 10 DS, 16 NF1, 15 cranial radiation, 7 congenital cardiac abnorality, 4renal artery stenosis	7.1 (6.0)	54 (37.8)
Shen et al. (2017) [65]	China	Case series	Fudan	2011 to 2014	134	77	IB–EDMS		6.5	38
Winstead et al. (2017) [66]	USA	Case series	Children’s Hospital and Research Center Oakland	2007	NR	7	IB–EDAS	7 MMS, 7 SCD	NR	2 (28.6)
Yang et al. (2017) [67]	USA	Case series	Johns Hopkins University School of Medicine	1990 to 2015	12	7	IB–EDAMS, EDAS, pial synangiosis	7 MMS, 7 SCD	6.9 (4.0)	4 (57.1)

*AS* alagille syndrome, *DB* direct bypass, *DS* Down syndrome, *EDAS* encephaloduroateriosynangiosis, *EDAMS* encephaloduroarteriomyosynangiosis, *EDMS* encephaloduromyosynangiosis, *EGS* encephalogaleosynangiosis, *EMAS* encephalomyoarteriosynangiosis, *EMS* encephalomyosynangiosis, *IB* indirect bypass, *MBH* multiple burr holes, *MMD* moyamoya disease, *MMS* moyamoya syndrome, *NF1* neurofibromatosis 1, *NR* not reported, *SCD* sickle cell disease, *STA-MCA* superficial temporal artery to middle cerebral artery bypass, *UK* United Kingdom, *USA* United States

*Unless otherwise stated. For pooling of means of numerical variables, we computed missing means and standard deviations (SDs) from medians, ranges (minimum to maximum), and interquartile ranges (IQRs) using the methods proposed by Hozo et al. and Wan et al.

Risk of bias assessment using the JBI checklist for cohort studies and case series are reported in Supplementary Tables S3 and S4.

### Baseline characteristics of patients

The gender of the patients was reported in 25 studies in a total of 1731 patients—45.0% male and 55.0% female. The mean and SD of their age were reported or imputable in 29 studies in a total of 2204 patients. Overall pooled mean age was 8.6 years (95% CI: 7.7; 9.5, *I*^2^ = 95.3% [*p* < 0.001]). In total, 308 patients had MMS. The pooled prevalence of MMS within the included population was 21.7% (95% CI: 1.1–86.9%, *I*^2^ = 54.2 [*p* < 0.001]). The number of patients with associated SCD, NF1, Down syndrome, cranial radiation, congenital cardiac abnormality, renal artery stenosis, ACTA2 mutation, and Alagille syndrome were 131 (42.5%), 47 (15.3%), 40 (13.0%), 24 (7.8%), 18 (5.8%), 6 (1.9%), 3 (1.0%), and 3 (1.0%), respectively.

### Perioperative adverse events

Table 2 presents a detailed summary of the pooled outcomes in each group and Table 3 presents a direct comparison of outcomes between the two groups. Table 4 summarizes the predictors of these outcomes identified on meta-regression.

**Table 2:** Pooled outcomes of included patients/hemispheres between the two groups (indirect bypass and direct/combined bypass)

Outcomes	Indirect bypass						Direct and combined bypass					
	No. of studies reporting variable	No. of patients/ hemispheres analyzed	Pooled proportion [95% confidence interval]	*I*^2^ (%)	P value of *I*^2^ (from *χ*^2^ test)	Quality of Evidence (GRADE)	No. of studies reporting variable	No. of patients/ hemispheres analyzed	Pooled proportion [95% confidence interval]	*I*^2^ (%)	*P* value of *I*^2^ (from *χ*^2^ test)	Quality of Evidence (GRADE)
Perioperative adverse events												
Perioperative seizures (hemispheres)	8	2473	0.84 [0.16; 4.26]	79.0	< 0.001	Low	2	140	0.00 [0.00; 1.00]	0.0	1.000	Low
Perioperative seizures (patients)	7	1060	1.32 [0.17; 9.38]	77.1	< 0.001	Low	NA	NA	NA	NA	NA	NA
Perioperative subdural hygroma (hemispheres)	3	44	4.55 [0.21; 51.74]	0.0	0.993	Low	NA	NA	NA	NA	NA	NA
Perioperative subdural hygroma (patients)	3	32	6.25 [0.29; 60.69]	0.0	0.996	Low	NA	NA	NA	NA	NA	NA
Perioperative extra-axial hemorrhage (hemispheres)	8	2592	1.53 [0.62; 3.75]	0.0	0.544	Low	NA	NA	NA	NA	NA	NA
Perioperative extra-axial hemorrhage (patients)	7	1126	4.09 [2.86; 5.80]	5.2	0.387	Low	NA	NA	NA	NA	NA	NA
Perioperative intracerebral hemorrhage (hemispheres)	4	1326	0.36 [0.01; 11.14]	62.3	0.047	Low	NA	NA	NA	NA	NA	NA
Perioperative intracerebral hemorrhage (patients)	4	656	0.46 [0.07; 2.82]	57.4	0.070	Low	NA	NA	NA	NA	NA	NA
Perioperative wound complication (hemispheres)	8	810	1.18 [0.31; 4.46]	54.1	0.033	Low	4	177	2.26 [0.46; 10.36]	0.0	0.584	Low
Perioperative wound complication (patients)	6	210	3.01 [0.61; 13.46]	41.0	0.132	Low	2	33	3.03 [0.00; 99.99]	0.0	1.000	Low
Perioperative CSF leak (hemispheres)	8	1245	1.00 [0.34; 2.89]	30.6	0.184	Low	2	125	1.60 [0.00; 99.29]	0.0	0.573	Low
Perioperative CSF leak (patients)	7	459	1.72 [0.39; 7.30]	37.7	0.141	Low	NA	NA	NA	NA	NA	NA
Perioperative hydrocephalus requiring shunt (hemispheres)	2	178	0.56 [0.00; 99.95]	0.0	1.000	Low	NA	NA	NA	NA	NA	NA
Perioperative hydrocephalus requiring shunt (patients)	2	110	0.91 [0.00; 99.97]	0.0	1.000	Low	NA	NA	NA	NA	NA	NA
Perioperative TIA (hemispheres)	16	1782	2.62 [1.14; 5.91]	67.8	< 0.001	Low	6	328	7.61 [2.20; 23.15]	78.8	< 0.001	Low
Perioperative TIA (patients)	16	753	4.52 [1.95; 10.09]	59.1	< 0.001	Low	3	106	9.74 [0.35; 76.75]	82.0	0.004	Low
Perioperative stroke (hemispheres)	24	3394	3.19 [1.91; 5.30]	54.8	< 0.001	Low	9	492	4.55 [2.04; 9.84]	53.1	0.030	Low
Perioperative stroke (patients)	20	1506	5.94 [3.74; 9.29]	26.1	0.138	Low	3	89	5.62 [0.81; 30.14]	0.0	0.905	Low
Perioperative death (patients)	20	1224	0.00 [0.00; 1.00]	0.0	1.000	Low	6	179	0.56 [0.04; 6.89]	0.0	1.000	Low
Outcomes at last follow-up												
Revascularization Matsushima Grade A (hemispheres)	14	822	56.70 [44.32; 68.29]	83.4	< 0.001	Low	5	284	44.40 [5.75; 91.27]	0.0	0.662	Low
Revascularization Matsushima Grades A and B (hemispheres)	14	822	85.61 [78.84; 90.48]	54.3	0.008	Low	5	284	95.42 [17.79; 99.95]	76.8	0.002	Low
Stroke recurrence at last follow-up (hemispheres)	9	1599	2.34 [0.88; 6.06]	64.8	0.004	Low	7	411	2.38 [0.39; 13.28]	0.0	0.996	Low
Stroke recurrence at last follow-up (patients)	16	1416	5.24 [2.97; 9.08]	54.6	0.005	Low	6	233	5.87 [1.41; 21.41]	0.0	0.890	Low
mRS0-1 at last follow-up (patients)	8	604	80.38 [68.67; 88.45]	81.0	< 0.001	Low	4	144	87.44 [39.85; 98.65]	0.0	0.734	Low
mRS2-3 at last follow-up (patients)	5	481	25.28 [3.97; 73.49]	39.8	0.156	Low	NA	NA	NA	NA	NA	NA
Mortality at last follow-up (patients)	18	1454	0.30 [0.08; 1.17]	0.0	1.000	Low	5	210	0.48 [0.03; 7.18]	0.0	1.000	Low

*NA* not applicable as fewer than 2 studies reported the outcome by hemisphere/patients

^*^When the pooled proportions (GLMM method) provided 95% CI of zero to one or nearly one, we advise to interpret with caution as the estimate is likely not reliable

**Table 3:** Direct comparison of outcomes between the two groups (indirect bypass and direct/combined bypass with indirect bypass as control)

**Outcomes**	**No. of studies reporting variable**	**No. of patients/hemispheres analyzed**	**Pooled effect size [95% confidence interval]**	***I***^**2**^** (%)**	***P* value of*I***^**2**^** (from*χ***^**2**^** test)**	**Quality of Evidence (GRADE)**
Perioperative wound complications (hemispheres)	2	582	RR 2.54 [1.82; 3.55]	0.0	0.978	Low
Perioperative seizures (hemispheres)	2	605	RR 0.25 [0.00; 2022.04]	0.0	0.514	
Perioperative TIA (hemispheres)	5	935	RR 0.64 [0.38; 1.10]	0.0	0.786	Low
Perioperative stroke (hemispheres)	6	1056	RR 1.04 [0.41; 2.65	18.9	0.290	Low
Total perioperative complications (hemispheres)	7	1056	RR 1.01 [0.86; 1.17]	1.2	0.415	Low
Perioperative death (hemispheres)	2	1162	RR 0.72 [0.00; 5682.31]	0.0	0.538	Low
Perioperative death (patients)	2	159	RR 0.96 [0.04; 22.76]	NA	NA	Low
Revascularization Matsushima Grade A (hemispheres)	3	144	RR 1.56 [0.99; 2.46]	0.0	0.707	Low
Revascularization Matsushima Grades A and B (hemispheres)	3	144	RR 1.12 [1.02; 1.24]	0.0	0.878	Low

**Table 4:** Predictors of outcome identified on meta-regression

**Outcome**	**No. of studies reporting outcome and risk factor**	**Total no. of patients/hemispheres analyzed**	**Predictor**	***P* value**
**Indirect**				
Perioperative stroke	24 24 24 24 24 24	3394 3394 3394 3394 3394 3394	Publication year Age Proportion of MMS Proportion of SCD Proportion of NF1 Proportion of DS	0.128 0.048 0.153 0.221 0.749 0.151
Perioperative TIA	16 16 16 16 16 16	753 753 753 753 753 753	Publication year Age Proportion of MMS Proportion of SCD Proportion of NF1 Proportion of DS	0.795 0.141 0.867 0.307 0.162 0.133
Revascularisation (Matsushima grades A and B)	14 14 14 14 14 14	822 822 822 822 822 822	Publication year Age Proportion of MMS Proportion of SCD Proportion of NF1 Proportion of DS	0.464 0.168 0.934 0.873 0.996 0.342
Stroke recurrence	16 16 16 16 16 16	1416 1416 1416 1416 1416 1416	Publication year Age Proportion of MMS Proportion of SCD Proportion of NF1 Proportion of DS	0.770 0.206 0.608 0.274 0.818 0.751
Mortality	18 18 18 18 18 18	1454 1454 1454 1454 1454 1454	Publication year Age Proportion of MMS Proportion of SCD Proportion of NF1 Proportion of DS	0.044 0.425 0.071 0.334 0.496 0.372
**Direct/combined**				
Perioperative stroke	9 8 4 NA 4 NA	492 438 383 NA 383 NA	Publication year Age Proportion of MMS Proportion of SCD Proportion of NF1 Proportion of DS	0.837 0.005 0.558 NA 0.357 NA
Perioperative TIA	6 6 NA NA NA NA	328 328 NA NA NA NA	Publication year Age Proportion of MMS Proportion of SCD Proportion of NF1 Proportion of DS	0.910 < 0.001 NA NA NA NA
Revascularisation (Matsushima grades A and B)	5 NA NA NA NA NA	284 NA NA NA NA NA	Publication year Age Proportion of MMS Proportion of SCD Proportion of NF1 Proportion of DS	0.057 NA NA NA NA NA
Stroke recurrence	7 5 NA NA NA NA	411 196 NA NA NA NA	Publication year Age Proportion of MMS Proportion of SCD Proportion of NF1 Proportion of DS	0.291 0.010 NA NA NA NA
Mortality	5 NA NA NA NA NA	210 NA NA NA NA NA	Publication year Age Proportion of MMS Proportion of SCD Proportion of NF1 Proportion of DS	0.566 NA NA NA NA NA

*NA* not applicable as there were too few studies for an accurate meta-regression

**Fig. 2:**
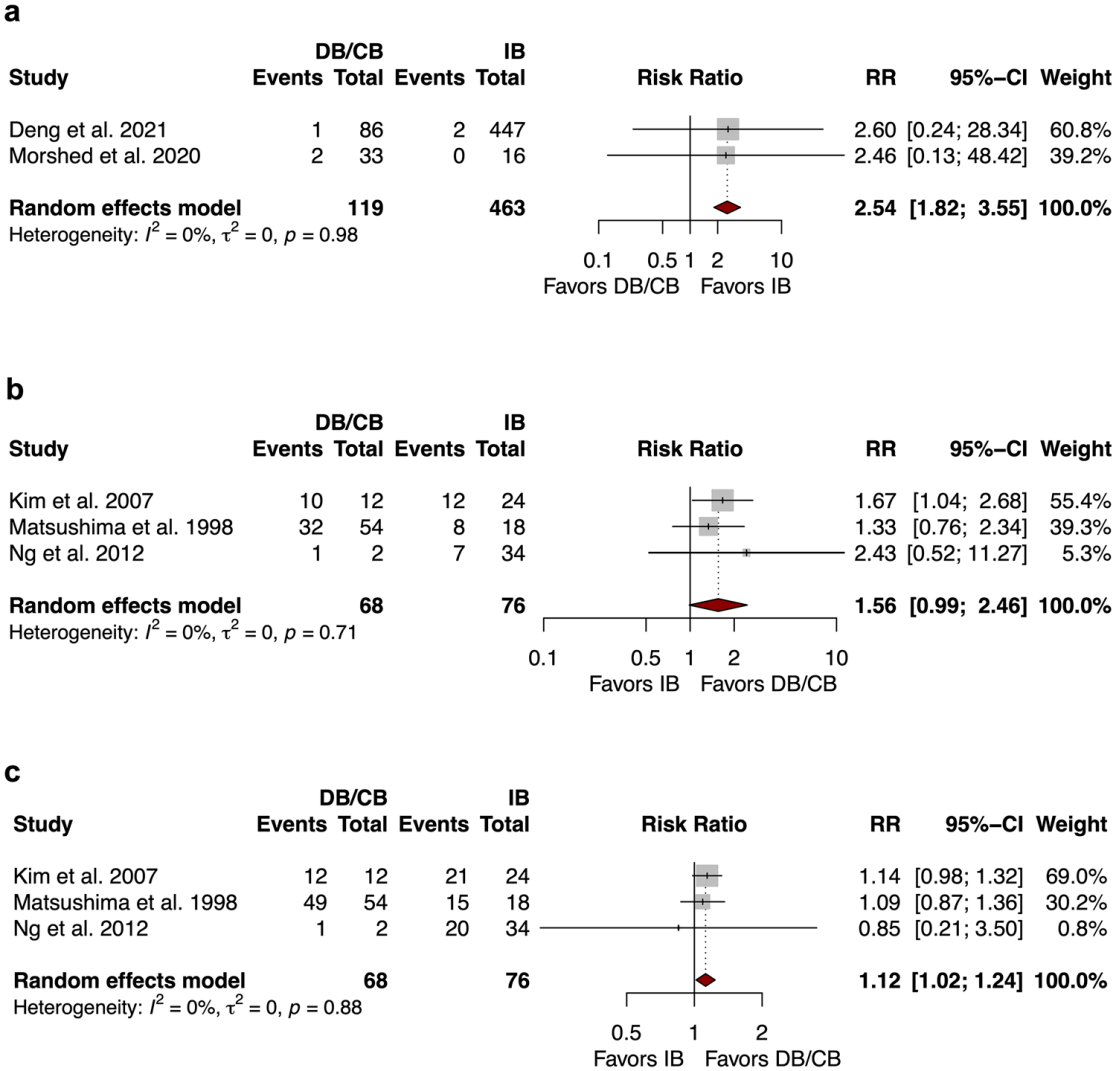
Forest plot comparing rates of **a** perioperative wound complication, **b** Matsushima grade A, and **c** Matsushima grade A/B between DB/CB versus IB

Overall pooled rates of perioperative seizures by hemispheres in the IB and DB/CB groups were 0.84% (95% CI: 0.16; 4.26, *I*^2^ = 79.0 [*p* < 0.001]) and 0.00% (95% CI: 0.00; 1.00, *I*^2^ = 0.0 [*p* = 1.000]) respectively. Two studies of 582 hemispheres directly compared rates of perioperative seizures between the two groups. Perioperative seizure rate was comparable between IB and DB/CB (RR = 0.25 (95% CI: 0.00; 2022.03), *I*^2^ = 0.0 [*p* = 0.514]). Overall pooled rates of perioperative wound complications by hemispheres in the IB and DB/CB groups were 1.18% (95% CI: 0.31; 4.46, *I*^2^ = 54.1 [*p* = 0.033]) and 2.26% (95% CI: 0.46; 10.36, *I*^2^ = 0.0 [*p* = 0.584]), respectively. Overall pooled rates of perioperative wound complications by patients in the IB and DB/CB groups were 3.01% (95% CI: 0.61; 13.46, *I*^2^ = 41.0 [*p* = 0.132]) and 3.03% (95% CI: 0.00; 99.99, *I*^2^ = 0.0 [*p* = 1.000]), respectively. Two studies of 582 hemispheres directly compared rates of perioperative wound complications between the two groups. Perioperative wound complications rate was significantly higher in the DB/CB group (RR = 2.54 (95% CI: 1.82; 3.55), *I*^2^ = 0.0 [*p* = 0978]) (Fig. 2a). Overall pooled rates of perioperative CSF leak by hemispheres in the IB and DB/CB groups were 1.00% (95% CI: 0.34; 2.89, *I*^2^ = 30.6 [*p* = 0.184]) and 1.6% (95% CI: 0.00; 99.29, *I*^2^ = 0.0 [*p* = 0.573]), respectively. No direct comparison was available for rates of perioperative CSF leaks.

**Fig. 3:**
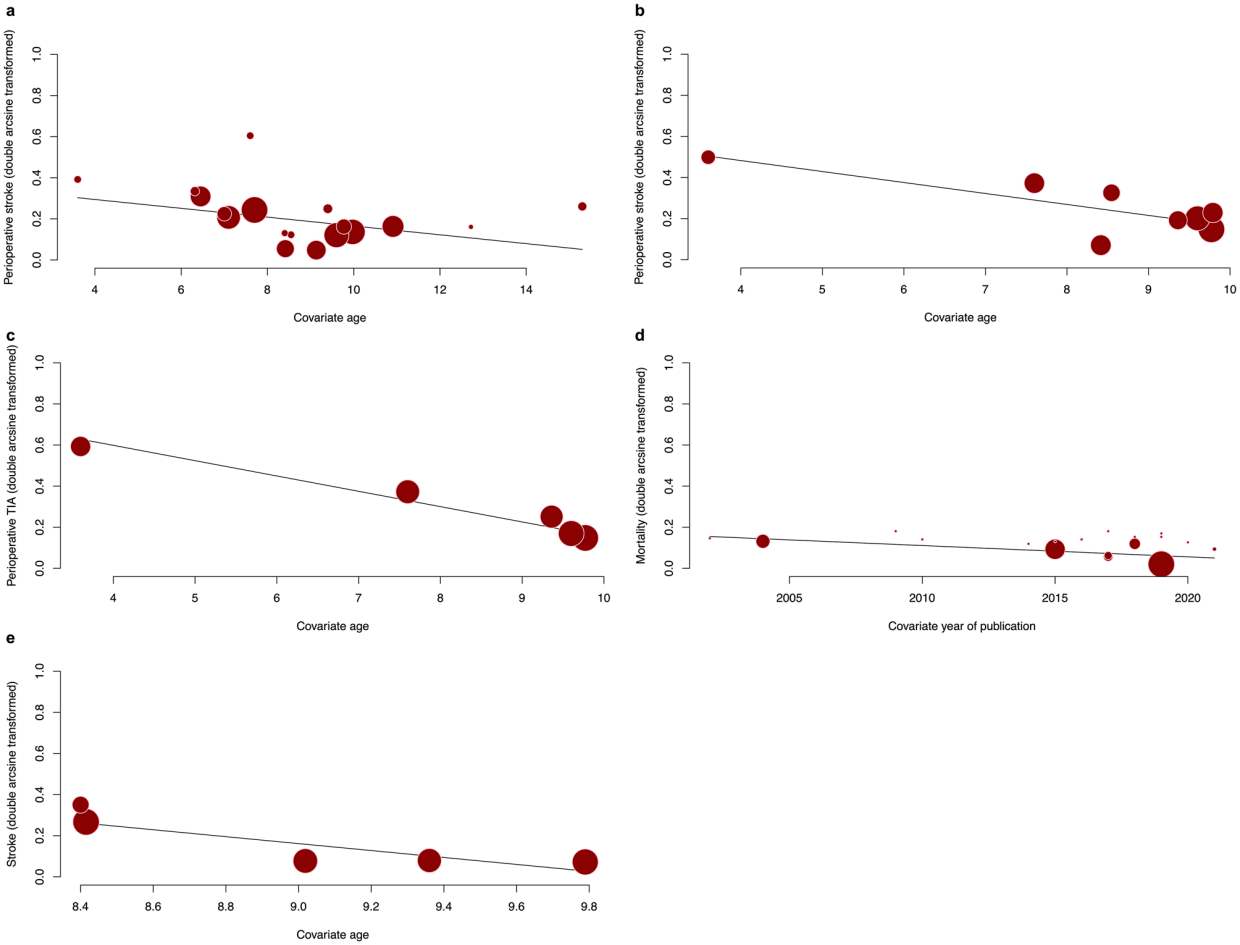
Bubble plot for meta-regression of transformed proportion of **a** perioperative stroke against age in each IB study, **b** perioperative stroke against age in each DB/CB study, **c** perioperative TIA against age in each DB/CB study, **d** mortality against year of publication in each IB study, and **e** transformed proportion of long-term stroke recurrence against age in each DB/CB study

Overall pooled rates of perioperative TIA by hemispheres in the IB and DB/CB groups were 2.62% (95% CI: 1.14; 5.91, *I*^2^ = 67.8 [*p* < 0.001]) and 7.61% (95% CI: 2.20; 23.15, *I*^2^ = 78.8 [*p* < 0.001]), respectively. Pooled rates of perioperative TIA by patients in the IB and DB/CB groups were 4.52% (95% CI: 1.95; 10.09, *I*^2^ = 59.1 [*p* < 0.001]) and 9.74% (95% CI: 0.35; 76.75, *I*^2^ = 82.0 [*p* = 0.004]), respectively. Five studies of 935 hemispheres directly compared rates of perioperative TIA. Perioperative TIA rate was comparable between IB and DB/CB (RR = 0.64 (95% CI: 0.38; 1.10), *I*^2^ = 0.0 [*p* = 0.786]). Pooled rates of perioperative stroke by hemispheres in the IB and DB/CB groups were 3.19% (95% CI: 1.915.30, *I*^2^ = 54.8 [*p* < 0.001]) and 4.55% (95% CI: 2.04; 9.84, *I*^2^ = 53.1 [*p* = 0.030]), respectively. Two studies directly compared rates of perioperative stroke by hemispheres and patients and showed comparability (RR = 0.25 (95% CI: 0.00; 2022.04), *I*^2^ = 0.0 [*p* = 0.514]) and (RR = 0.72 (95% CI: 0.00; 5682.31), *I*^2^ = 0.0 [*p* = 0.538]), respectively. On meta-regression, age significantly predicted rates of perioperative stroke (*p* = 0.048) in the IB group (Fig. 3a). Further meta-regression demonstrated age further significantly predicted rates of perioperative TIA (*p* = 0.005) and perioperative stroke (*p* < 0.001) in the DC/CB group (Fig. 3b and c, respectively).

Pooled rates of perioperative death in the IB and DB/CB groups were 0.00% (95%CI: 0.00; 1.00, *I*^2^ = 0.0 [*p* = 1.000]) and 0.56% (95%CI: 0.00; 6.89, *I*^2^ = 0.0 [*p* = 1.000]), respectively. Direct comparison between the groups showed comparability (RR = 0.96 (95% CI: 0.04; 22.76), *I*^2^ = NA [*p* = NA]).

### Revascularization

Angiographical follow-up duration was reported in 9 studies comprising 1150 hemispheres and pooled duration was 4.3 years (95% CI: 2.2; 6.4, *I*^2^ = 99.5 [*p* < 0.001]).

In the IB group, overall pooled rates of hemispheres with Grade A and Grade A/B revascularization were 56.70% (95% CI: 44.32; 68.29, *I*^2^ = 83.4 [*p* < 0.001]) and 85.61% (95% CI: 78.84; 90.48, *I*^2^ = 54.3 [*p* = 0.008]). In DB/CB group, overall pooled rates of hemispheres with Grade A and Grade A/B revascularization were 44.44% (95% CI: 5.75; 91.27, *I*^2^ = 0.0 [*p* = 0.662]) and 95.42% (95% CI: 17.79; 99.95, *I*^2^ = 76.8 [*p* = 0.002]). Three studies of 144 hemispheres directly compared proportions of Grade A and Grade A/B revascularization. No significant difference in the proportion of Grade A was identified (RR = 1.56 (95% CI 0.99; 2.46), *I*^2^ = 0.0 [*p* = 0.707]), but proportions of Grade A/B favored DB/CB over IB (RR = 1.12 (95% CI 1.02; 1.24), *I*^2^ = 0.0 [*p* = 0.878]) (Fig. 2b and c).

### Stroke recurrence, dependence, and mortality at last follow up

Clinical follow-up duration was reported in 33 studies with a total of 1992 patients and pooled duration was 6.5 years (95% CI: 4.4; 8.6, *I*^2^ = 99.0 [*p* < 0.001]).

Overall pooled rates of stroke recurrence by hemispheres at last follow-up in the IB and DB/CB groups were 2.34% (95% CI: 0.88; 6.06, *I*^2^ = 64.8 [*p* = 0.004]) and 2.38% (95% CI: 0.39; 13.28, *I*^2^ = 0.0 [*p* = 0.996]), respectively. Overall pooled rates of stroke recurrence by patients at last follow-up in the IB and DB/CB groups were 5.24% (95% CI: 2.97; 9.08, *I*^2^ = 51.6 [*p* = 0.005]) and 5.87% (95% CI: 1.41; 21.41, *I*^2^ = 0.0 [*p* = 0.890]), respectively. On meta-regression, age (*p* = 0.010) significantly predicted stroke recurrence in the DC/CB group (Fig. 3e).

Overall pooled rates of patients with mRS scores of 0 and 1 at last follow-up in the IB and DB/CB groups were 80.38% (95% CI: 68.67; 88.45, *I*^2^ = 81.0 [*p* < 0.001]) and 87.44% (95% CI: 39.85; 98.65, *I*^2^ = 0.0 [*p* = 0.734]), respectively. Overall pooled rates of mortality at last follow-up in the IB and DB/CB groups were 0.30% (95% CI: 0.08; 1.17, *I*^2^ = 0.0 [*p* = 1.000]) and 0.48% (95% CI: 0.03; 7.18, *I*^2^ = 0.0 [*p* = 1.000]), respectively. The year of publication (*p* = 0.044) significantly predicted mortality in the IB group (Fig. 3d).

No direct comparison between the two groups was available for rates of stroke recurrence, dependence, or mortality at last follow-up.

## Discussion

### Summary of findings

This study represents an accurate systematic review and meta-analysis investigating the role of IB, DB, and CB in pediatric patients with MMD/MMS. Both IB and DB/CB procedures had evidence of efficacy and low rates of complication. A comparative meta-analysis demonstrated a significant benefit in favor of DB/CB in terms of long-term angiographic outcomes, when compared with IB; however, wound complication rates were higher following DB/CB. Other outcomes including perioperative seizures, TIA, stroke, and death were similar between the two groups.

### In comparison with the literature

The paucity of studies reporting on DB/CB and widespread available studies investigating IB reflects current patterns of practice favoring IB in the pediatric MMD population. While EDAS and EDAMS were among the originally described techniques for IB, new techniques such as pial synangiosis and multiple burr holes have been added to the surgical armamentarium [38]. Existing evidence is insufficient for there to be consensus regarding the optimal IB technique.

This meta-analysis found low rates of perioperative complications in both DB/CB and IB groups. When compared with IB, CB/DB has been purported to be more technically challenging with a greater risk for postoperative complications [49]. However, many studies have demonstrated the feasibility and safety of DB/CB in pediatric patients with satisfactory outcomes [3, 21, 43, 49]. Factors dissuading the use of DB/CB over IB in the pediatric MMD population, include smaller-caliber recipient and donor vessels, the potential for cross-clamp-induced ischemia, and the risk of poor scalp wound healing. This latter concern was substantiated by the findings of this metanalysis [4, 43]. The lower rates of perioperative adverse events, ranging from wound complications to ischemic events, in our meta-analysis may in general, reflect improved patient selection, anesthetic, and peri-operative care with further knowledge into the management of pediatric MMD/MMS [8]. Regardless of the technique, revascularization should in general be performed in high-volume centers as there is evidence to suggest that caseload correlates with improved care and reduced mortality in pediatric patients with MMD/MMS [68].

In a recent meta-analysis comparing the three bypass techniques in adults, Nguyen et al. [69] found that DB/CB conferred benefits in terms of late stroke recurrence versus IB, with no dissimilarities in terms of perioperative outcomes. Notably, while cerebral hyperperfusion is an undesirable complication of DB in adult patients, this phenomenon is much less frequently observed in pediatric patients and so the conclusions of this study should not constitute a reason to avoid DB in children.

This current metanalysis found evidence of improved angiographic outcomes following DB/CB in comparison with IB; a finding in accordance with previous meta-analyses [70]. Jeon et al. [70] additionally demonstrated a significantly lower risk of future stroke events for DB compared with IB in symptomatic adult patients, although we failed to find evidence of this benefit in our pediatric population.

It has been suggested that patients with various subtypes of MMS undergoing revascularization have poorer outcomes when compared with cases with MMD [5, 7, 19, 20, 59]. Lack of stratification between treatment groups did not allow for a comparison of revascularization strategies between these two pathologies in this current analysis. Furthermore, our meta-regression did not identify the presence of MMS nor its specific phenotypes to significantly affect outcomes; however, this is likely be a function of the limited number of studies reporting them, leading to a Type 2 error. Our meta-regression analysis, however, did identify younger age to be associated with a higher risk of peri-operative stroke and TIA complications. This is consistent with the literature which suggests that younger children with MMD/MMS are thought to be the most severely affected and most challenging to treat [8]. This is likely due to their dynamic clinical course, leading to major strokes on presentation, and poor eventual outcomes [8]. Infants with MMD/MMS have severely compromised cerebrovascular reserve and are particularly vulnerable to anesthetic risks [8].

### Clinical implications

As this meta-analysis was not able to directly compare IB and DB/CB for all the stated outcomes, we can at best conclude that both techniques are comparable except for the association of greater rates of angiographic revascularization and wound complication rates in DB/CB. Based on this meta-analysis, it would be prudent to counsel families that although DB/CB is associated of greater rates of angiographic revascularization, this does not necessarily translate into any additional benefit over IB in terms of clinical outcomes such perioperative TIA, perioperative stroke, and long-term stroke recurrence. Indeed, certain studies have suggested a poor correlation between Grades A/B revascularization and future stroke risk [59]. DB/CB allows for immediate augmentation of cerebral blood flow and does not rely on the plasticity and angiogenic potential, unlike IB. In contrast to the immediate flow augmentation by the anastomosis of DB/CB, IB generally relies on the slow neovascularization and recruitment of collaterals over time. In this respect, angiographic success with DB/CB is more reflective of technical anastomosis success. Due to this, the interpretation of angiographic outcomes from IB may be limited if the time to collateral angiogenesis is inadequate, which may explain the findings of our study.

### Implication on the direction of future research in MMD/MMS intervention

This systematic review underlines the inconsistency in measurement and reporting within the literature of MMD/MMS. Several included primary studies had not distinguished their outcomes based on the type of bypass, patient population (adult vs pediatric), nor whether or not outcomes were reported in terms of hemispheres or patients. Indeed, previous meta-analyses have also encountered this predicament [10, 11]. This inconsistency in reporting impedes data aggregation and outcome comparison across studies, hindering progress in MMD/MMS management. Conducting a randomized controlled trial in pediatric patients with a rare progressive disease such as MMD/MMS is near impossible due to ethical reasons [1, 2], which highlights the urgency and need for greater standardization in reporting. Consistent reporting in MMD/MMS can be facilitated by an agreed minimum set of indicators to be reported. With a unified standard of data reporting, this will enable valid evidence syntheses and ultimately implementation of management recommendations.

### Limitations

Limitations of this meta-analysis include the retrospective and observational nature of included studies. Our study has also highlighted the limited number of studies directly comparing DB/CB and IB for MMD/MMS. This could explain the finding of non-significance in the various outcomes. Additionally, apart from perioperative events, there were no standard time frame with different lengths of clinical follow-up in each study. Furthermore, several outcomes reported in this study have a large encompassing confidence interval, which may be explained by the modest sample size and large heterogeneity between studies. As such, we advise to interpret these outcomes with great caution as the estimates were unlikely to be reliable. Only studies published in English were included; therefore, selection bias may exist because MMD has greater incidence rates among Asian populations. Based on the information from the included studies, our current meta-analysis could not assess whether or not the translation of subjective angiographic assessments across grading scales were accurate in the pediatric cohort. A possible relationship may be uncovered in future with more granular detail. Validation can be achieved by establishing a prospective data registry collected from multiple international centers which can inform future individual participant data meta-analysis in real-world settings. Our meta-analysis included a diverse range of patients of various ethnic diversity, enhancing its external validity. The large number of studies enabled us to perform a meta-regression to explore possible confounders. However, we cannot exclude the possibility that the conclusions drawn in our study may have been affected by residual confounders. Confounders that we did not control for in our analyses include surgeon experience although we controlled for the year of publication given that the surgical and peri-operative management of these patients has generally improved over time due to greater accrued understanding of the condition with time. Most importantly, this meta-analysis is the most reliable and transparent to date as we excluded repeated patient populations from the same institutions within overlapping time intervals.

## Conclusions

IB, DB/CB techniques have both been demonstrated to be effective and safe revascularization options for pediatric MMD/MMS. A paucity of cohort studies has a limit direct comparison between these interventions. Available low-quality GRADE evidence suggests that DB/CB is associated with better long-term revascularization outcomes when compared with IB alone, although this did not translate to better long-term stroke outcomes.

## Supplementary Information

Below is the link to the electronic supplementary material.

Supplementary file1 (TIFF 272235 KB)

Supplementary file2 (DOCX 40 KB)

Supplementary file3 (DOCX 32 KB)

## Data Availability

Not applicable.
