# Supplementary material for: Surgical revascularizations for pediatric moyamoya: a systematic review, meta-analysis, and meta-regression analysis
Source: Childs Nerv Syst. 2023 Feb 8;39(5):1225–43. doi: 10.1007/s00381-023-05868-6 (PMC10167165; doi:10.1007/s00381-023-05868-6)
Supplement: Supplementary file 2 — Supplementary file2 (DOCX 40 KB) [file 381_2023_5868_MOESM2_ESM.docx]

Supplementary Table 1. Full search phrases used for the 3 respective databases on 7^th^ October 2022.

| **Ovid MEDLINE** | | | 521 articles |
| --- | --- | --- | --- |
| **Moyamoya disease/syndrome concept** | | | |
| 1 | exp Moyamoya disease/ | | |
| 2 | moyamoya.tw | | |
| 3 | 1 or 2 | | |
| **Surgery concept** | | | |
| 4 | exp Cerebral Revascularization/ or revascular*.tw or revascularization.tw or revascularisation.tw or exp Anastomosis, Surgical/ or anastomos*.tw or bypass.tw or direct.tw or indirect.tw or combined.tw | | |
| 5 | exp Temporal Arteries/ or superficial temporal arter*.tw or STA.tw or exp Middle Cerebral Artery/ or MCA.tw or STA-MCA.tw or STAMCA.tw | | |
| 6 | encephaloduroarteriosynangiosis.tw or encephalo-duro-arterio-synangiosis.tw or EDAS.tw or encephaloduroarteriomyosynangiosis.tw or encephalo-duro-arterio-myo-synangiosis.tw or EDAMS.tw or pial synangiosis.tw or synangiosis.tw or burr holes.tw | | |
| 7 | 4 or 5 or 6 | | |
| **Pediatric concept** | | | |
| 8 | exp pediatrics/ or pediatric.tw. or paediatric.tw. or children.tw. | | |
| **Combined concepts** | | | |
| 9 | 3 and 7 and 8 | | |
| 10 | limit 9 to english language | | |
| **Embase** | | | 939 articles |
| **Moyamoya disease/syndrome concept** | | | |
| 1 | exp Moyamoya disease/ | | |
| 2 | moyamoya.tw | | |
| 3 | 1 or 2 | | |
| **Surgery concept** | | | |
| 4 | exp Cerebral Revascularization/ or revascular*.tw or revascularization.tw or revascularisation.tw or exp Anastomosis/ or anastomos*.tw or exp Brain Artery Bypass/ or exp bypass surgery/ or bypass.tw or direct.tw or indirect.tw or combined.tw | | |
| 5 | exp Temporal Artery/ or superficial temporal arter*.tw or STA.tw or exp Middle Cerebral Artery/ or MCA.tw or STA-MCA.tw or STAMCA.tw | | |
| 6 | encephaloduroarteriosynangiosis.tw or encephalo-duro-arterio-synangiosis.tw or EDAS.tw or encephaloduroarteriomyosynangiosis.tw or encephalo-duro-arterio-myo-synangiosis.tw or EDAMS.tw or pial synangiosis.tw or synangiosis.tw or burr holes.tw | | |
| 7 | 4 or 5 or 6 | | |
| **Pediatric concept** | | | |
| 8 | exp pediatrics/ or pediatric.tw. or paediatric.tw. or children.tw. | | |
| **Combined concepts** | | | |
| 9 | 3 and 7 and 8 | | |
| 10 | limit 9 to english language | | |
| **Cochrane Controlled Register of Trials CENTRAL** | | 28 articles | |
| **Moyamoya concept** | | | |
| 1 | MeSH descriptor: [Moyamoya Disease] explode all trees | | |
| 2 | (“Moyamoya”):ti,ab,kw | | |
| 3 | #1 OR #2 | | |
| **Surgery concept** | | | |
| 4 | MeSH descriptor: [Cerebral Revascularization] explode all trees | | |
| 5 | MeSH descriptor: [Anastomosis, Surgical] explode all trees | | |
| 6 | MeSH descriptor: [Temporal Arteries] explode all trees | | |
| 7 | MeSH descriptor: [Middle Cerebral Artery] explode all trees | | |
| 8 | (superficial temporal arter* or STA or MCA or STA-MCA or STAMCA):ti,ab,kw | | |
| 9 | (encephaloduroarteriosynangiosis or encephalo-duro-arterio-synangiosis or EDAS or encephaloduroarteriomyosynangiosis or encephalo-duro-arterio-myo-synangiosis or EDAMS or pial synangiosis or synangiosis or burr holes):ti,ab,kw | | |
| 10 | #5 OR #6 OR #7 OR #8 OR #9 OR #10 | | |
| **Combined concepts** | | | |
| 11 | #3 AND #10 | | |

Supplementary Table 2. Inclusion and exclusion criteria used to select studies for the review

| Inclusion criteria | Exclusion criteria |
| --- | --- |
| Primary interventional or observational studies assessing the effectiveness and safety of revascularization in children (aged <18 years) with Moyamoya disease/syndrome | - Not written in English - Systematic reviews and meta-analysis, editorials, commentaries, opinion papers, letters, education papers, conference abstracts, protocols, reports, theses or book chapters - Treatment not tested in the clinical setting (e.g. lab based rather than clinical practice) - Did not report outcomes of interests, or meaningful extractable data - Did not report outcomes that can be assigned to pediatric patients - Did not report outcomes that can be assigned to specific bypass procedures - Overlapping populations - Arm <5 patients |

Supplementary Table S3. Joanna Briggs Institute quality assessment checklist for cohort studies

|  |  | | **Question no.** | | | | | | | | | |  | |  | |
| --- | --- | --- | --- | --- | --- | --- | --- | --- | --- | --- | --- | --- | --- | --- | --- | --- |
| **Study** | **1** | **2** | | **3** | **4** | **5** | **6** | **7** | **8** | **9** | **10** | **11** | | **Overall** | |  |
| Araki et al. 2022 | ✔ | ✔ | | ✔ | ✔ | ✔ |  | ✔ | ✔ | ✔ | ✔ | ✔ | | 10 | |  |
| Deng et al. 2021 | ✔ | ✔ | | ✔ | ✔ | ✔ |  | ✔ | ✔ | ✔ | ✔ | ✔ | | 10 | |  |
| Guzman et al. 2009 | ✔ | ✔ | | ✔ | ✔ | ✔ |  | ✔ | ✔ | ✔ | ✔ | ✔ | | 10 | |  |
| Ishikawa et al. 1997 | ✔ | ✔ | | ✔ |  |  |  | ✔ | ✔ | ✔ | ✔ | ✔ | | 8 | |  |
| Kim et al. 2007 | ✔ | ✔ | | ✔ | ✔ | ✔ |  | ✔ | ✔ | ✔ | ✔ | ✔ | | 10 | |  |
| Matsushima et al. 1998 | ✔ | ✔ | | ✔ |  |  |  | ✔ | ✔ | ✔ | ✔ | ✔ | | 8 | |  |
| Morshed et al. 2020 | ✔ | ✔ | | ✔ | ✔ | ✔ |  | ✔ | ✔ | ✔ | ✔ | ✔ | | 10 | |  |
| Sadashiva et al. 2016 | ✔ | ✔ | | ✔ |  |  |  | ✔ | ✔ | ✔ | ✔ | ✔ | | 8 | |  |

1. Were the two groups similar and recruited from the same population?

2. Were the exposures measured similarly to assign people to both exposed and unexposed groups?

3. Was the exposure measured in a valid and reliable way?

4. Were confounding factors identified?

5. Were strategies to deal with confounding factors stated?

6. Were the groups/participants free of the outcome at the start of the study (or at the moment of exposure)?

7. Were the outcomes measured in a valid and reliable way?

8. Was the follow up time reported and sufficient to be long enough for outcomes to occur?

9. Was follow up complete, and if not, were the reasons to loss to follow up described and explored

10. Were strategies to address incomplete follow up utilized?

11. Was appropriate statistical analysis used?

Supplementary Table S4. Joanna Briggs Institute quality assessment checklist for case series.

|  |  | | **Question no.** | | | | | | | | |  |
| --- | --- | --- | --- | --- | --- | --- | --- | --- | --- | --- | --- | --- |
| **Study** | **1** | **2** | | **3** | **4** | **5** | **6** | **7** | **8** | **9** | **10** | **Overall** |
| Alamri et al. 2019 | ✔ | ✔ | | ✔ | ✔ | ✔ | ✔ | ✔ | ✔ | ✔ | ✔ | 10 |
| Bao et al. 2015 | ✔ | ✔ | | ✔ | ✔ | ✔ | ✔ | ✔ | ✔ | ✔ | ✔ | 10 |
| Blauwblomme et al. 2017 | ✔ | ✔ | | ✔ | ✔ | ✔ | ✔ | ✔ | ✔ | ✔ | ✔ | 10 |
| Chen et al. 2018 | ✔ | ✔ | | ✔ | ✔ | ✔ | ✔ | ✔ | ✔ | ✔ | ✔ | 10 |
| Czabanka et al. 2009 | ✔ | ✔ | | ✔ | ✔ | ✔ | ✔ | ✔ | ✔ | ✔ | ✔ | 10 |
| Darwish et al. 2005 | ✔ | ✔ | | ✔ | ✔ | ✔ | ✔ |  |  | ✔ | ✔ | 8 |
| De Oliveira et al. 2009 | ✔ | ✔ | | ✔ | ✔ | ✔ | ✔ |  |  | ✔ | ✔ | 8 |
| Funaki et al. 2014 | ✔ | ✔ | | ✔ | ✔ | ✔ | ✔ | ✔ | ✔ | ✔ | ✔ | 10 |
| Furtado et al. 2021 | ✔ | ✔ | | ✔ | ✔ | ✔ | ✔ | ✔ | ✔ | ✔ | ✔ | 10 |
| Gadgil et al. 2018 | ✔ | ✔ | | ✔ | ✔ | ✔ | ✔ | ✔ | ✔ | ✔ | ✔ | 10 |
| Goren et al. 2021 | ✔ | ✔ | | ✔ | ✔ | ✔ | ✔ | ✔ | ✔ | ✔ | ✔ | 10 |
| Griessenauer et al. 2015 | ✔ | ✔ | | ✔ | ✔ | ✔ | ✔ | ✔ | ✔ | ✔ | ✔ | 10 |
| Ha et al. 2019 | ✔ | ✔ | | ✔ | ✔ | ✔ | ✔ | ✔ | ✔ | ✔ | ✔ | 10 |
| Hall et al. 2016 | ✔ | ✔ | | ✔ | ✔ | ✔ | ✔ | ✔ | ✔ | ✔ | ✔ | 10 |
| Isono et al. 2002 | ✔ | ✔ | | ✔ | ✔ | ✔ | ✔ | ✔ | ✔ | ✔ | ✔ | 10 |
| Karasawa et al. 1992 | ✔ | ✔ | | ✔ | ✔ | ✔ |  |  |  | ✔ | ✔ | 7 |
| Kennedy et al. 2014 | ✔ | ✔ | | ✔ | ✔ | ✔ | ✔ | ✔ | ✔ | ✔ | ✔ | 10 |
| King et al. 2010 | ✔ | ✔ | | ✔ | ✔ | ✔ | ✔ | ✔ | ✔ | ✔ | ✔ | 10 |
| Kuroda et al. 2010 | ✔ | ✔ | | ✔ | ✔ | ✔ | ✔ | ✔ | ✔ | ✔ | ✔ | 10 |
| Mirone et al. 2019 | ✔ | ✔ | | ✔ | ✔ | ✔ | ✔ | ✔ | ✔ | ✔ | ✔ | 10 |
| Ng et al. 2012 | ✔ | ✔ | | ✔ | ✔ | ✔ | ✔ | ✔ | ✔ | ✔ | ✔ | 10 |
| Ogiwara et al. 2012 | ✔ | ✔ | | ✔ | ✔ | ✔ | ✔ | ✔ | ✔ | ✔ | ✔ | 10 |
| Ong et al. 2020 | ✔ | ✔ | | ✔ | ✔ | ✔ | ✔ | ✔ | ✔ | ✔ | ✔ | 10 |
| Rashad et al. 2016 | ✔ | ✔ | | ✔ | ✔ | ✔ | ✔ | ✔ | ✔ | ✔ | ✔ | 10 |
| Sadashiva et al. 2016 | ✔ | ✔ | | ✔ | ✔ | ✔ | ✔ | ✔ |  | ✔ | ✔ | 9 |
| Sakamoto et al. 1997 | ✔ | ✔ | | ✔ | ✔ | ✔ | ✔ |  |  | ✔ | ✔ | 7 |
| Scott et al. 2004 | ✔ | ✔ | | ✔ | ✔ | ✔ | ✔ | ✔ | ✔ | ✔ | ✔ | 10 |
| Shen et al. 2017 | ✔ | ✔ | | ✔ | ✔ | ✔ | ✔ | ✔ | ✔ | ✔ | ✔ | 10 |
| Winstead et al. 2017 | ✔ | ✔ | | ✔ | ✔ | ✔ | ✔ | ✔ |  |  | ✔ | 8 |
| Yang et al. 2017 | ✔ | ✔ | |  |  | ✔ | ✔ | ✔ | ✔ | ✔ | ✔ | 8 |

1. Were there clear criteria for inclusion in the case series?

2. Was the condition measured in a standard, reliable way for all participants included in the

case series?

3. Were valid methods used for identification of the condition for all participants included in the

case series?

4. Did the case series have consecutive inclusion of participants?

5. Did the case series have complete inclusion of participants?

6. Was there clear reporting of the demographics of the participants in the study?

7. Was there clear reporting of clinical information of the participants?

8. Were the outcomes or follow up results of cases clearly reported?

9. Was there clear reporting of the presenting site(s)/clinic(s) demographic information?

10. Was statistical analysis appropriate?
